# Dental manifestations of hypophosphatasia in children and the effects of enzyme replacement therapy on dental status: A series of clinical cases

**DOI:** 10.1002/ccr3.2769

**Published:** 2020-03-13

**Authors:** Larisa Kiselnikova, Elena Vislobokova, Victoria Voinova

**Affiliations:** ^1^ Pediatric Dentistry Department A.I. Yevdokimov Moscow State University of Medicine and Dentistry Moscow Russia; ^2^ Department of Clinical Genetics Research and Clinical Institute for Pediatrics Named After Academician Yuri Veltischev Pirogov Russian National Research Medical University Moscow Russia

**Keywords:** asfotase alfa, dental signs, enzyme replacement therapy, hypophosphatasia, premature loss of primary teeth

## Abstract

The most frequent dental signs of hypophosphatasia in children are premature loss of primary teeth, decrease in height of alveolar bone, and malocclusions. Enzyme replacement therapy with Asfotase alfa might be associated with stabilization of dental status.

## Graphical abstract

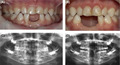

## 1 INTRODUCTION

The study included 16 patients (1‐17 years old) with hypophosphatasia. The most frequent dental signs were premature loss of primary teeth (43.8%), decrease in height of alveolar bone (43.8%), and various malocclusions (62.5%). Asfotase alfa replacement therapy (2 mg/kg subcutaneously three times a week) in 10 patients lasting 1.6‐3.9 years was associated with stabilization of dental status.

Hypophosphatasia (HPP, OMIM 146300, 241500, 241510) is a progressive hereditary metabolic disorder that is characterized by a deficit of tissue nonspecific alkaline phosphatase (ALP) due to mutations in the gene encoding this enzyme (*ALPL*; OMIM# 171760).^1^ *ALPL* gene mutations are identified in 94% of HPP cases.^2^ Currently, at least 392 distinct mutations have been revealed, and the majority of them (70.2%) are missense mutations.^2^ ALP is present in the liver, bones, and kidneys as well as in dentin, dental cementum, and alveolar bones of the jaws.^1^ HPP can be inherited in either autosomal‐dominant or autosomal‐recessive patterns.^2^ The clinical severity varies widely from intrauterine fetal death due to impaired bone mineralization, up to premature loss of teeth without any musculoskeletal disorders.^1^ Autosomal‐recessive forms of HPP are characterized by an early manifestation and are the most severe form of the disease, whereas autosomal‐dominant forms are clinically milder.^2^ The prevalence of severe HPP forms is 1:300 000 in Europe and 1:100 000 in Canada.^2^ In Russia, the expected prevalence is 1:100 000.^3^

A decrease in ALP activity disrupts the decay of its substrates that are accumulated in biological fluids. ALP cleaves inorganic pyrophosphate to phosphate in the bone tissues that is required for hydroxyapatite formation. Hydroxyapatite is the main mineral component of the skeleton. In early childhood, mineral metabolism disorders associated with HPP lead to bone hypomineralization, osteomalacia, and fractures.^1^, ^2^

The same factor interferes with mineralization of the organic matrix of hard dental tissues (dentin and cementum), which affects tooth attachment to the alveoli.^4^ The main dental sign of HPP is a premature loss of primary teeth resulting in persistent morphological and functional alterations in the child's masticatory system and the need for further rehabilitation of masticatory function. Such clinical cases are often described in the published literature.^5^, ^6^ In contrast, loss of dentoalveolar attachment, pathologic tooth mobility, or loss of permanent teeth are rarely seen in patients with HPP.^2^, ^7^

Currently, the only effective treatment option for HPP is enzyme replacement therapy with asfotase alfa.^6^, ^8^ Published data demonstrate significant success in using this drug for the treatment of the most severe forms of HPP (prenatal and infantile) and describe the increase in bone mineral density and improvement in respiratory and motor functions.^6^, ^8^, ^9^ Some authors advise prescription of asfotase alfa to patients with the childhood form of HPP and odontohypophosphatasia, who were carefully selected by evaluating the severity of signs and symptoms, the expected benefit, and the potential risks.^10^ The effects of enzyme replacement therapy on the dental status of patients have not yet been studied.

Therefore, the aim of the present study was to evaluate the dental signs of HPP in children and the need for dental treatment, as well as the effects of enzyme replacement therapy with asfotase alfa on dental status.

## 2 METHODS

This prospective observational study included a nonrandom sample of all patients with HPP aged 0‐18 years who were referred for dental examination by the pediatric clinic. The duration of the observation ranged from 1.6 to 3.9 years (median 2.4 years).

The Simplified Oral Hygiene Index (OHI‐S, Greene JC, Vermillion JR)^11^ was used to assess oral hygiene status, and the papillary‐marginal‐alveolar index (PMA, Parma C., 1960) was used to assess the periodontium status. Dental panoramic radiography/cone beam computed tomography (CBCT) was used to identify potential anomalies in the teeth or jaw and to evaluate the alveolar bone status. To identify anomalies in tooth formation, all teeth, except for the third molars, were examined.

For each patient, the need for dental care was assessed, the individual treatment plan was developed, and preventive instructions were provided. The patients received medical treatment depending on their dental and general health status. The main objectives of the dynamic dental follow‐up and treatment of patients with HPP included the following: to decrease the microbial factors playing a major role in the progression of periodontal abnormalities involving the loss of tooth attachment, to restore masticatory function, and to prevent secondary deformations due to a premature loss of teeth.

## 3 RESULTS

The prospective observations included 16 patients aged 14 months to 17 years with *ALPL* gene mutations and biochemical signs of HPP. The baseline characteristics of the observed patients are shown in Table 1. The majority (15 out of 16) of patients were referred for dental examination by the pediatric clinics. In 1 case, the patient's parents applied for dental treatment on their own initiative.

**Table 1:** Baseline characteristics of patients with HPP under observation (n = 16)

Patient	Form of HPP	Dental signs	Age, years	Sex	Plasma ALP level, IU/L	Age‐related reference range^a^ for plasma ALP level, IU/L	ALP to lower limit of normal ratio, %	Asfotase alfa treatment
1	Infantile	Yes	2.5	M	17	142‐335	12.0	Yes
2 (case 2)	Infantile	Yes	5.5	F	40	142‐335	28.2	Yes
3 (case 3)	Infantile	Yes	10	F	63	129‐417	48.8	Yes
4	Infantile	No	5.5	F	69	96‐297	71.9	Yes
5	Infantile	No	2	F	107	156‐369	68.6	Yes
6	Childhood	Yes	6	M	86	142‐335	60.6	No
7	Childhood	Yes	10	M	90	129‐417	69.8	No
8	Childhood	Yes	8	M	88	142‐335	62.0	No
9 (case 1)	Childhood	Yes	1.2	M	NA^b^			No
10 (case 4)	Childhood	Yes	3	M	74	142‐335	52.1	Yes
11	Childhood	Yes	17	M	18	50‐150	36.0	Yes
12	Childhood	No	9	M	104	142‐335	73.2	Yes
13	Childhood	No	4	M	109	142‐335	76.8	Yes
14	Childhood	No	5.5	M	86	142‐335	60.6	Yes
15	Childhood	No	5	M	119	142‐335	83.8	No
16	Odontohypophosphatasia	Yes	17	F	48	50‐150	96.0	No

Abbreviations: ALP, alkaline phosphatase; HPP, hypophosphatasia.

^a^ The presented data are based on medical records available before the treatment. Different methods of ALP assessment were used.

^b^ No available data at the time of referral, the diagnosis of HPP was confirmed later.

The infantile form of HPP was diagnosed in five patients, 10 patients had a childhood form, and 1 patient had odontohypophosphatasia. In 6 cases, patients’ relatives were carriers of *ALPL* gene mutations. Fifteen patients had a low plasma ALP level at the time of referral, and in 1 case, the diagnosis of HPP was confirmed later. Ten out of 16 patients were assigned to enzyme replacement therapy with asfotase alfa by the pediatricians.

The following rachitic deformities in the musculoskeletal system were observed in 11 patients: frontal bossing, rachitic rosary, varus or valgus deformations of the lower extremities, and scoliosis, as well as joint hypermobility and low muscle tone. Six out of 16 children had neurologic disorders, such as delayed psychomotor and speech development, epilepsy, autism, and seizures.

Premature loss of primary teeth was documented in 7 out of 16 examined children. The average age of tooth eruption was 6‐8 months, and then, at the age of 12‐14 months, the teeth started to fall out. The first teeth to become mobile and fall out were generally the lower and upper frontal teeth, followed by the more distant from the central line teeth. The root formation of the shed teeth was not completed. Parents reported a lack of bleeding during shedding of the teeth.

Dental examination revealed caries lesions in 11 children (7 children had rampant caries—DMF index more than 6).

The majority of patients had mild gingivitis (PMA index—16%‐32%) and poor oral hygiene levels (OHI index—2.0‐2.8). One child had severely abundant supragingival mineralized deposits. Malocclusions, dental arch anomalies, and irregular teeth position were observed in 10 patients.

Below are the clinical cases of HPP with the most striking dental signs.

### 3.1 Case 1

**Figure 1:**
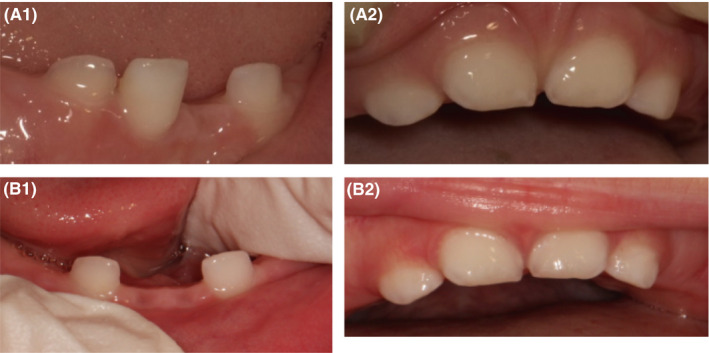
Case 1, childhood form of HPP. Clinical view: (A) at 14 mo of age; (B) at 16 mo of age

A 14‐month‐old boy was referred by his pediatric dentist for spontaneous and unexplained loss of his primary lower left central incisor. The patient's history reported normal growth. Except for the absence of the central lower left incisor and extreme mobility of the lower right central incisor, the oral examination was entirely normal, without inflammation of the gums (Figure 1). Thus, the child was referred to a geneticist for the diagnosis of suspected HPP. Genetic analysis revealed a mutation in the *ALPL* gene and confirmed the diagnosis of the childhood form of HPP. The second examination at 2 months revealed the absence of the lower right central incisor. The father reported that the tooth fell out without bleeding.

Premature loss of primary teeth, as the most typical sign of HPP, allowed the disease to be diagnosed at the early stage during the primary dental examination of a 14‐month‐old patient.

### 3.2 Case 2

A 4‐year‐old female patient came from the Endocrinology Research Centre for a dental examination. Infantile form HPP had been recently diagnosed. Since the diagnosis, the patient had received enzyme replacement therapy with asfotase alfa (Strensiq). There was no family history of metabolic or genetic disorders. The mother complained that many of the primary teeth of her daughter had spontaneously fallen out. At the age of 11 months, the lower central incisors erupted, and at 12 months, they were shed. At the age of 20 months, the lower lateral incisors fell out. By the age of 2 years, the upper central incisors had fallen out. At 3 years and 6 months, the lower canines were shed. Nevertheless, the mother reported no history of trauma.

**Figure 2:**
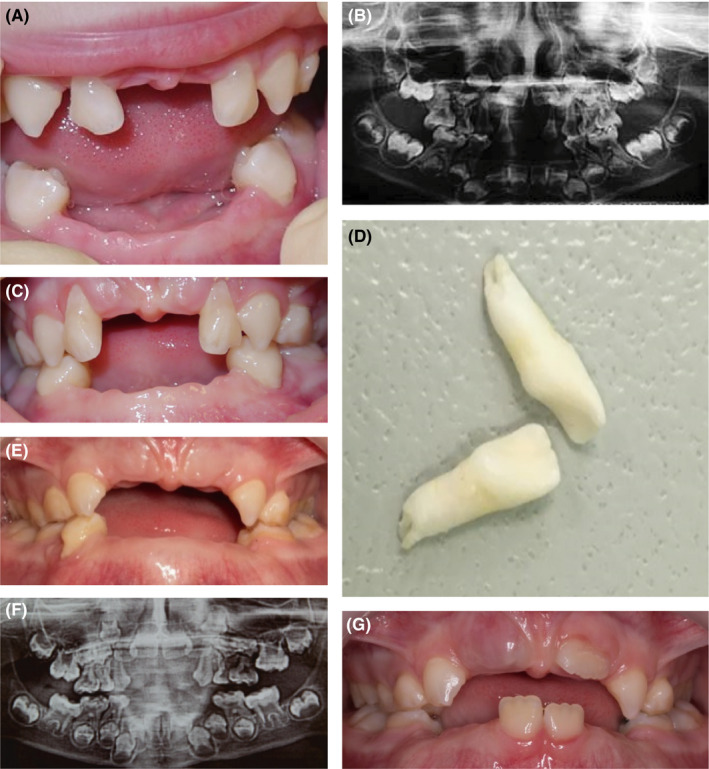
Case 2, infantile form of HPP. Clinical view and panoramic radiograph: (A, B) at the age of 4 y; (C) at the age of 4.5 y; (E, F) at the age of 6 y; (G) at the age of 7.5 y; (D) lower incisors that fell out at the age of 2 y

Physical examination revealed short stature, a bulging frontal bone, lower limb bow, and. Intraoral examination revealed an upper arch with the absence of primary central incisors and caries‐free primary dentition (Figure 2). The primary lateral upper incisors were Grade 2 mobile. There was minimal gingival inflammation. The lower arch exhibited the absence of primary frontal teeth due to premature shedding. The upper lateral incisors and the first lower left molar were lost spontaneously because of extreme mobility despite the start of enzyme replacement therapy. Radiographically, enlarged pulp chambers and shape abnormalities of the permanent teeth crowns were revealed. Horizontal alveolar bone loss reached nearly half of the root length.

The patient received asfotase alfa (2 mg/kg subcutaneously three times a week) for more than 3 years. At the last clinical examination, the authors registered a significant improvement and a complete lack of mobility of the remaining primary teeth.

### 3.3 Case 3

This 10‐year‐old girl was diagnosed with the infantile form of HPP. Despite the fact that the patient presented signs of HPP from birth, the diagnosis was only established at the age of 2 years, following the loss of her anterior primary teeth. At 6 months, primary teeth began to erupt, and 6 months later, they began to fall out. The roots of the fallen teeth were not formed.

The patient received asfotase alfa (2 mg/kg subcutaneously three times a week) for more than 3 years.

**Figure 3:**
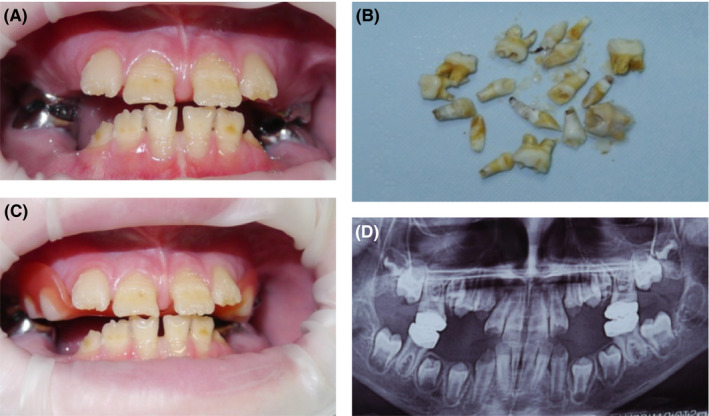
Case 3, infantile form of HPP: (A) clinical view at the age of 10 y: enamel hypoplasia of all the erupted teeth; the first permanent molars were restored by standard stainless‐steel crowns; (B) premature loss of primary teeth with unformed roots and enamel hypoplasia; (C) clinical view with a replacement laminar denture in the oral cavity; (D) panoramic radiograph at the age of 10 y

Intraoral examination revealed enamel hypoplasia with exposure of the dentin of the lower and upper permanent front teeth (Figure 3). All first permanent molars were particularly affected and restored by stainless‐steel crowns. The mobility of teeth and periodontal tissue pathology were not noted. X‐ray examination revealed a significantly reduced alveolar bone height in the area of missing primary molars.

A denture was fabricated to restore the patient's function and prevent space problems in the dental arch.

### 3.4 Case 4

The next case is a 2.5‐year‐old male patient in whom the therapy was effective in reducing primary teeth mobility. His mother was worried about the spontaneous loss of his lower primary central incisors with an interval of 2 weeks at the age of 12 months. The initial value of serum ALP was low (74 IU/L), and a diagnosis of HPP was considered by his pediatrician. Genetic analysis revealed 2 mutations in the ALPL gene. The patient was diagnosed with the childhood form of HPP.

**Figure 4:**
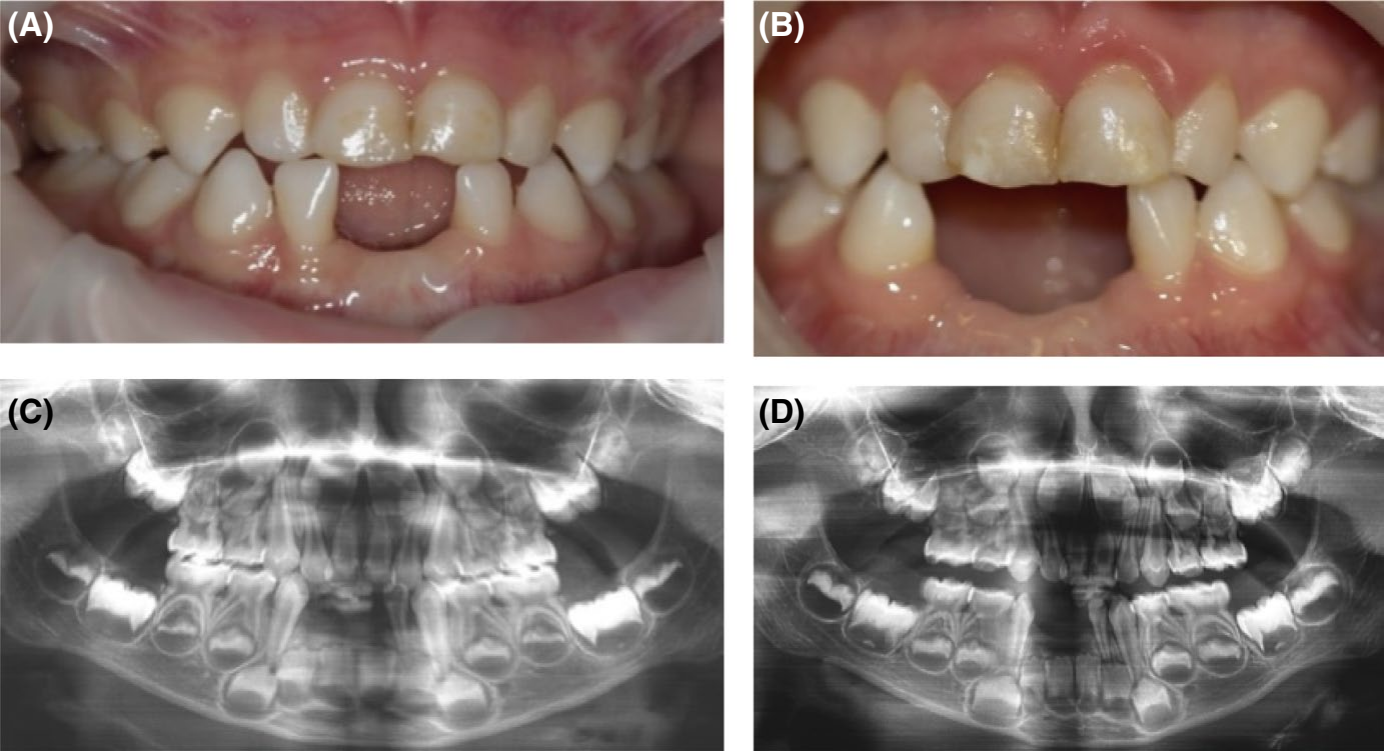
Case 4, childhood form of HPP. Clinical view and panoramic radiograph: (A, C) at the age of 2.5 y: absence of prematurely lost lower central incisors; (B, D) at the age of 4.5 y: absence of 3 lower incisors, recession of the gingival margin in the area of the upper incisors

Intraoral examination revealed a lower arch with caries‐free, incomplete primary dentition and absence of lower central incisors (Figure 4), right lateral lower incisor with Grade II mobility, and upper central incisors with Grade I mobility.

Since the diagnosis, the patient received therapy with asfotase alfa (2 mg/kg subcutaneously three times a week).

The second examination in 2 years revealed the absence of a right lateral lower incisor. Despite recession of the gingival margin, the mobility of the upper central incisors decreased significantly.

The most common dental signs of HPP in the studied cohort included (Table 2) a premature loss of primary teeth with completed and uncompleted root formation (43.8%) or their mobility (37.5%), as well as a decrease in the alveolar bone height (43.8%) and various malocclusions (62.5%). Other typical signs included enamel hypoplasia (25%) and enlarged pulp chambers and root canals (25%). No cases of affected tooth attachment to the alveoli, pathological mobility, or loss of permanent teeth were observed.

**Table 2:** Rate of dental signs in patients with HPP

Dental signs, n	Primary dentition (1‐6 y of age), n = 7	Transitional dentition (6‐12 y of age), n = 6	Permanent dentition (12 y of age and older), n = 3	Total, n = 16	Rate, %
Premature loss of primary teeth	3	3	1	7	43.8
Tooth mobility	3	3	0	6	37.5
Enamel hypoplasia	1	2	1	4	25.0
Enlarged pulp chamber and root canals	0	3	1	4	25.0
Resorption of alveolar bone tissue	3	3	1	7	43.8
Recession of the gingival margin	1	2	0	3	18.8
Malocclusions	3	5	2	10	62.5

The average plasma ALP level among the patients with dental signs of HPP constituted 51.7% of the age‐specific lower limit standard, whereas the patients with no clinical dental signs had 72.5% of the age‐specific lower limit standard (Table 1). In the majority of cases, patients with lower levels of ALP had more severe dental signs of HPP.

The patients underwent the following therapeutic and preventive procedures: professional oral hygiene, training of children, and their parents that included the selection of individual oral hygiene items depending on the severity of the general condition; recommendations on the use of fluoride‐containing dental toothpastes, xylitol‐containing tooth wipes, remineralizing therapy, and sealing of the molar and premolar fissures; and treatment of caries and enamel hypoplasia using various dental sealing materials and stainless‐steel crowns. For children with premature loss of primary teeth, dentures were fabricated.

One of the study objectives included the investigation of the effects of enzyme replacement therapy on the dental status of the patients. During the observation, none of the patients receiving asfotase alfa (2 mg/kg subcutaneously three times a week) developed premature mobility of previously stable primary teeth, which indicated process stabilization. In 1 case, the patient who started treatment at the age of 2.5 years demonstrated a significant decrease in the mobility of the primary upper central incisors despite the remaining recession of the gingival margin.

On the other hand, another child (case 2) demonstrated a clinically defined improvement in the muscle tone and motor functions, as identified by the endocrinologist, but continued to lose primary teeth despite the ongoing enzyme replacement therapy prescribed at the age of 4 years. The authors believe that the continued loss of teeth at the start of the treatment cycle was due to severe damage of the periodontal ligament of those teeth and the irreversibility of the pathological changes. After the loss of primary teeth with mobility Grades II and III, stabilization was achieved. More than 3 years after the start of the therapy, none of the remaining primary teeth was mobile.

## 4 DISCUSSION

The decrease in alkaline phosphatase activity associated with HPP in early childhood causes inadequate bone mineralization and organic matrix of the hard dental tissues (dentin and cementum), which affects tooth attachment to the alveoli. The main dental sign of the disease is a premature loss of primary teeth.

The obtained results correlated with published data. Premature loss of primary teeth is considered to be the most typical dental sign of HPP.^4^, ^6^, ^12^ Other common characteristics include enlarged pulp chambers and enamel hypoplasia. Tooth eruption disorders, such as late tooth eruption and ankylosis of primary teeth, can also be observed.^12^

During the study course, there were no cases of affected tooth attachment to the alveoli, pathological mobility, or loss of permanent occlusion. Several publications contain limited data about the damage of permanent teeth in patients with HPP.^5^, ^7^, ^13^ Four patients were observed to have various degrees of enamel hypoplasia of the permanent teeth. Similar amelogenesis impairment of the permanent teeth in patients with HPP has also been described by Bloch‐Zupan A.^14^

Premature loss of primary teeth allowed the authors to diagnose HPP at the early stage during a primary dental examination of a 14‐month‐old patient and to timely refer the patient for further examination, which confirmed the diagnosis. Some authors emphasize the important role of a dentist in the primary diagnosis of HPP.^15^

The authors noted a certain correlation between the severity of dental disorders and a decrease in plasma ALP levels. In the majority of cases, the more severe the observed dental signs of HPP were, the less were low levels of ALP relative to the age‐specific lower limit standard noted. Similar results were published by Reibel A.^12^

None of the patients receiving asfotase alfa suffered from premature mobility of previously stable primary teeth. In some cases, the teeth with mobility Grade I became stable during the treatment, and no mobility was observed in those teeth during the follow‐up, although recession of the gingival margin was present. Thus, the process of premature loss of primary teeth in children receiving enzyme replacement therapy with asfotase alfa was stabilized. However, the efficacy of asfotase alfa therapy for the dental health of HPP patients requires further study. Whyte MP also noted that prior to the enzyme replacement therapy prescription, the potential risks and expected benefit of the treatment should be considered, taking into account HPP form and severity.^6^

It was observed that the age when the patient started the enzyme replacement therapy was also important for dental sign correction. Timely assignment to asfotase alfa treatment and an adequate enzymatic level in early childhood can contribute to the normal formation of the dental cementum and periodontal tissues, preventing anomaly development.

The limitations of the current investigation in relation to the asfotase alfa efficacy evaluation included an open‐label and noncomparative study design and a small number of enrolled patients (which nonetheless was significant for such a rare disease).

The goal of this publication is to raise awareness among dentists of the possible signs of HPP, which will improve early diagnosis of this disease because premature loss of primary teeth can be the only visible manifestation of HPP.

## CONFLICT OF INTEREST

The authors declare no conflicts of interest.

## AUTHOR CONTRIBUTIONS

LK: conceived the ideas and contributed to the design of the study. EV: examined and treated patients, and collected and analyzed the data. VV: contributed to interpretation of data. All authors contributed to the writing, critically reviewed, and approved the final version of the manuscript.
